# Antiviral effect of vitamin A on norovirus infection via modulation of the gut microbiome

**DOI:** 10.1038/srep25835

**Published:** 2016-05-16

**Authors:** Heetae Lee, GwangPyo Ko

**Affiliations:** 1 Center for Human and Environmental Microbiome, Institute of Health and Environment, School of Public Health, Seoul National University, Gwanak-ro, Gwanak-gu, Seoul 151-742, Korea; 2 N-BIO, Seoul National University, Gwanak-ro, Gwanak-gu, Seoul 151-742, Korea

## Abstract

The effect and underlying mechanism of vitamin A on norovirus infection are largely unknown. This study aimed to investigate how vitamin A administration affects the gut microbiome after norovirus infection. Here, we demonstrate that treatment with either retinol or retinoic acid (RA) inhibits murine norovirus (MNV) replication using both *in vitro* and *in vivo* models. Compositional changes in the gut microbiome associated with RA administration and/or norovirus infection were also investigated. Oral administration of RA and/or MNV significantly altered intestinal microbiome profiles. Particularly, bacterial species belonging to the Lactobacillaceae families were remarkably increased by MNV inoculation and RA administration, suggesting that the antiviral effects of RA occur via the modulation of specific microbiota. The antiviral causal effect of *Lactobacillus* was identified and demonstrated using *in vitro* models in RAW264.7 cells. The antiviral immune response to MNV was mediated by IFN-β upregulation. This study represents the first comprehensive profiling of gut microbiota in response to RA treatment against norovirus infection. Moreover, we conclude that the abundance of *Lactobacillus* through gut microbiota modulation by RA is at least partially responsible for norovirus inhibition.

Norovirus is the most frequent etiological viral agent of acute gastroenteritis among all age groups worldwide. Norovirus causes approximately 90% of all epidemic nonbacterial outbreaks of gastroenteritis around the world, and is responsible for approximately 50% of all foodborne outbreaks of gastroenteritis in the United States and many other countries^1^,^2^. Viral infection causes various clinical symptoms, including diarrhea, vomiting, nausea, abdominal pain, and fever lasting one to three days. Unfortunately, there is no current treatment or vaccine effective against norovirus infection.

A previous epidemiological study suggested that vitamin A supplementation decreases norovirus infection rates and clinical symptoms^3^. Moreover, the responses of various intestinal cytokines were modified by vitamin A supplementation during norovirus infection^4^. Retinoic acid (RA), the metabolite of dietary vitamin A, contributes to both innate and adaptive immune responses^5^. Additional evidence also indicates that RA deficiency impairs immunity, whereas RA excess can induce inflammatory disorders^5^. Retinoic acid-inducible gene 1 (RIG-1) and melanoma differentiation-associated gene 5 (MDA5) signaling play crucial roles in antiviral responses to viral RNA by producing type I interferons (IFNs)^6^. Moreover, a recent study reported that sufficient vitamin A supplementation reduced both mortality and morbidity associated with infectious gastrointestinal and respiratory diseases^7^.

The gut microbiota plays a pivotal role in pathogen infection and mucosal immune responses through cross-talk with mucosal immune systems^8^,^9^. For example, an altered gut microbiome in mice lacking Toll-like receptors (TLRs) and myeloid differentiation primary response gene 88 (Myd88) was strongly associated with metabolic syndrome, type 1 diabetes (T1D) and host defense against microbial infection^10^,^11^,^12^,^13^. Above all, dietary foods and drugs play a crucial role in modulating gut microbiome diversity, with changes that are directly linked to health conditions. For example, recent studies suggested that *Akkermansia muciniphila,* which increased significantly in the gut environment as a result of metformin treatment, may improve metabolic diseases such as type 2 diabetes^14^,^15^,^16^.

In this study, we evaluated: 1) compositional changes in the gut microbiota and host immune responses following RA treatment, and 2) the anti-norovirus effects of specific gut microbiota whose abundance was increased by RA treatment (*Lactobacillus* spp.).

## Results

### Effect of vitamin A on norovirus replication *in vitro* and *in vivo*

**Figure 1:**
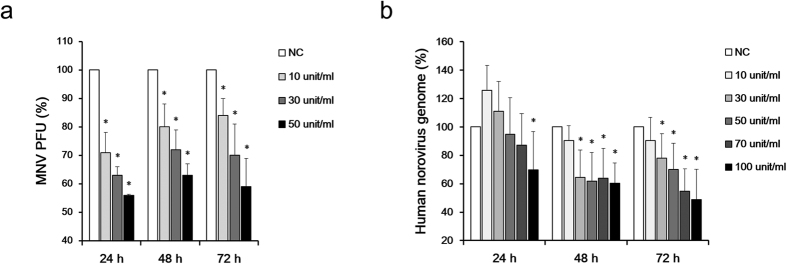
Treatment with vitamin A effectively inhibited norovirus replication *in vitro*. (**a**) Inhibition of MNV replication by retinol treatment. MNV (MOI 0.01) was infected into RAW 264.7 cells. Cells were then treated with retinol, and MNV replication was measured using a plaque assay. (**b**) Expression of the human norovirus genome. HG23 cells were incubated with various concentrations of retinol for 24–72 h. Total RNA of the human norovirus genome was quantified by real-time RT-PCR. *In vitro* tests were repeated three times. Significance was analyzed by the Mann–Whitney U-test and compared to the negative control. *p < 0.05. Retinol did not exhibit cytotoxicity under the conditions used in this experiment.

To confirm whether vitamin A inhibits norovirus replication, we first tested the inhibitory effect of retinol on MNV replication in murine RAW 264.7 cells. Retinol treatment (10, 30 and 50 U/mL) significantly inhibited MNV plaque formation at 24, 48 and 72 h (Fig. 1a). A similar inhibitory effect was observed for human norovirus genome replication by monitoring its effect on human norovirus replicon-bearing cells at 24, 48 and 72 h following retinol treatment (Fig. 1b). Twenty-four hours after retinol treatment, the copy number of the human norovirus genome had decreased significantly in the presence of 100 U/ml retinol compared to the negative control. At 48 and 72 h, retinol concentrations above 30 U/mL exhibited a significant inhibitory effect. The effect of RA was assessed based on the experimental infection of ICR mice with MNV, which induced a subclinical infection but no weight loss. Based on these infection conditions, ~60% of MNV-infected mice had cleared MNV at 72 h post-infection, indicating that MNV could establish a persistent infection in approximately 40% of infected mice without RA treatment. In contrast, the rate of MNV persistence in MNV-infected mice decreased to 28.6% in mice treated with 1 mg/kg/day RA.

### Analysis of the gut microbiome

**Figure 2:**
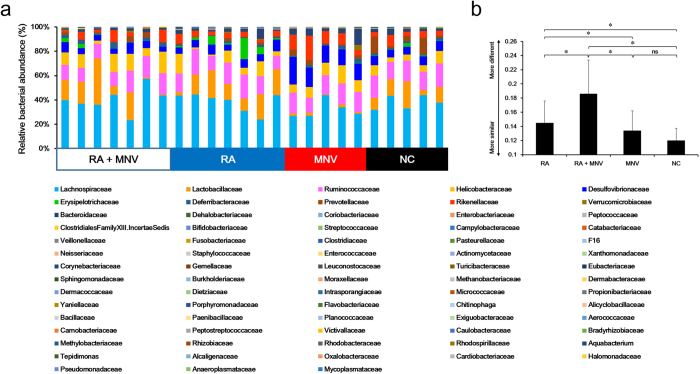
Taxonomic comparison based on 16S rRNA genes in a cecum sample. RA administration (RA) (n = 7) and MNV inoculation (n = 5) significantly altered the composition of the gut microbiome compared to the negative control (n = 5). In particular, MNV inoculation significantly altered the composition of the gut microbiota during RA administration (n = 7), and significantly changed bacterial abundances in cecum samples. (**a**) Relative bacterial abundance was classified at the family level. (**b**) UniFrac distance in groups categorized by RA administration and MNV inoculation. Samples from the MNV-inoculated group following RA administration showed the most significant differences in bacterial community composition. Statistically significant differences between groups were determined by the Mann–Whitney U-test. *p < 0.05.

To understand the presumed perturbation in the gut microbiome by either RA administration or MNV infection, a total of 2,670,123 sequences were generated after quality filtering 29 mouse cecum samples. Collected sequences were subsequently analyzed and classified into the following 13 prokaryotic phyla: Actinobacteria, Bacteroidetes, Cyanobacteria, Deferribacteres, Euryarchaeota, Firmicutes, Fusobacteria, Lentisphaerae, Proteobacteria, SR1, Tenericutes, TM7, and Verrucomicrobia. Figure 2a shows the relative gut microbiome abundance at the family level. RA treatment and MNV administration resulted in significantly different bacterial communities (Fig. 2b).

### Characteristics of the gut microbiome following RA administration

**Figure 3:**
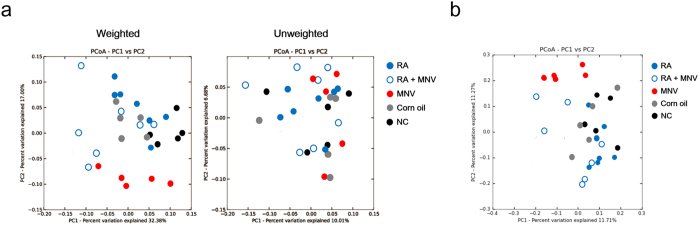
Microbial diversity by retinoic acid (RA) administration and MNV inoculation. (**a**) Beta diversity in groups categorized by RA administration and MNV inoculation was assessed by weighted and unweighted principle coordinate analysis (PCoA). Five groups categorized by RA administration and MNV inoculation were clearly clustered in the weighted, but not the unweighted, analysis. This result indicated that the abundances of certain bacterial taxa were changed by MNV inoculation following RA administration. (**b**) PCoA of KEGG pathways predicted by PICRUSt. MNV inoculation and RA administration also affected the KEGG pathway categories, as well as the bacterial diversity.

**Figure 4:**
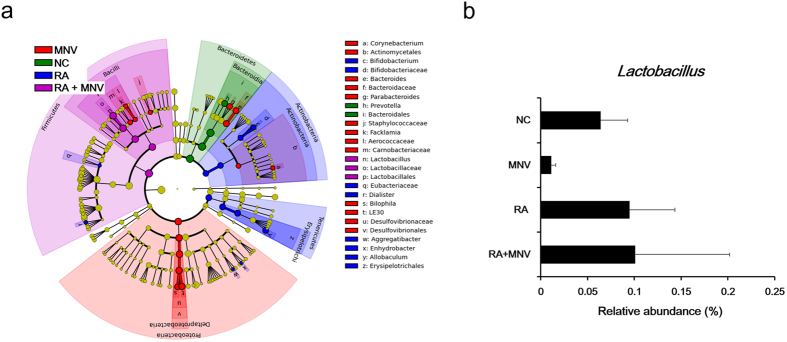
Significant bacterial abundances according to RA administration and MNV inoculation. (**a**) Characterization of bacterial abundance by MNV and RA. Significant differences were identified by LEfSe analysis as a *p* value <0.05 in both the Kruskal–Wallis test (among classes) and Wilcoxon test (between subclasses). The threshold logarithmic LDA score was 3.0. NC: negative control. RA was suspended in corn oil, and administered orally. (**b**) Abundance of *Lactobacillus* by RA administration and MNV infection. Among abundant bacteria from the LEfSe analysis, the abundance of *Lactobacillus* is shown separately. Bacterial abundances were decreased by MNV infection and increased by RA administration after MNV inoculation.

Seven samples from the RA-administered group were clustered in principle coordinate analysis (PCoA) of weighted UniFrac distances (Fig. 3a). Bacterial *Allobaculum, Aggregatibacter, Bifidobacterium, Dialister*, and *Enhydrobacter* genera were significantly increased in mice treated with RA (Fig. 4a). Moreover, KEGG pathways predicted by PICRUSt did not show clear clustering by RA administration or bacterial beta diversity (Fig. 3b).

### Characteristics of the gut microbiome following MNV infection

In contrast to the effect of RA administration, MNV infection during RA administration significantly affected the gut microbiome. When MNV was infected during RA administration, seven samples were clearly clustered into groups based on beta diversity of the gut microbiome (Fig. 3a). Microbiome diversity was greater in PCoA analysis of weighted UniFrac than unweighted. Figure 3a shows the relative bacterial abundance following MNV inoculation during RA administration. After MNV inoculation, Lactobacillaceae populations were significantly decreased compared to those in negative control (NC) mice (Fig. 4b). Furthermore, the *Lactobacillus* genus was significantly increased when MNV was inoculated during RA administration compared to other groups. Clustering into five groups was observed for PCoA data based on functional KEGG pathways (Fig. 3b). In addition, among the predicted KEGG pathways, the counts for glycolysis/gluconeogenesis (carbohydrate metabolism), benzoate degradation (xenobiotic biodegradation and metabolism), ABC transporters (membrane transport), the phosphotransferase system (PTS) (membrane transport), and signal transduction mechanisms (cellular processes and signaling) were significantly altered by MNV inoculation during RA administration compared with those that underwent MNV inoculation only.

### Inhibition of MNV replication by *Lactobacillus* strains *in vitro*

**Figure 5:**
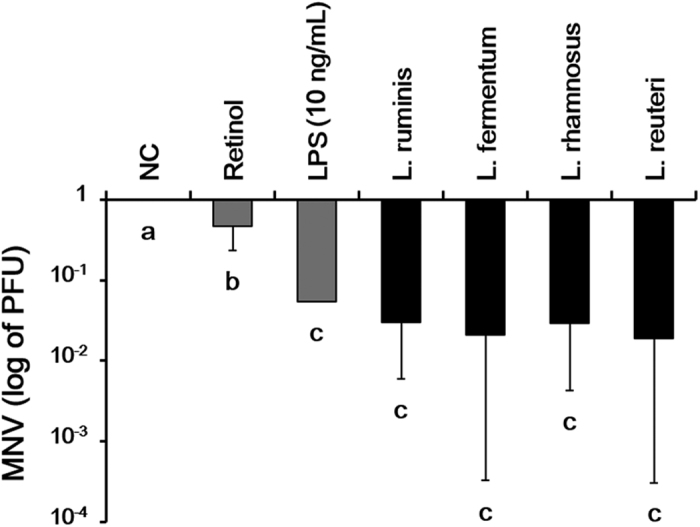
Inhibition of MNV replication by *Lactobacillus* strains in RAW264.7 cells. MNV replication was significantly inhibited by four *Lactobacillus* strains and retinol (50 U/mL). *Lactobacillus* (estimated at 1 × 10^5^ CFU by OD) was inoculated into MNV-inoculated RAW 264.7 cells (0.01 MOI) for 24 h in the absence of retinol. All *Lactobacillus* strains significantly decreased the replication of MNV. LPS (10 ng/mL), used as a control, showed an antiviral effect similar to that of *Lactobacillus*. After freezing and thawing twice, a plaque assay was performed to quantify MNV. Different superscript letters indicate significant differences (*p* < 0.05) according to Duncan’s *post hoc* test.

Four *Lactobacillus* strains (*L. ruminis* SPM 1308, *L. fermentum* KCTC 3112, *L. rhamnosus* KCTC 18427P, and *L. reuteri* KCTC 18428P) showed a significant inhibitory effect on MNV replication compared to retinol treatment alone (10^−1^ to 10^−2^, Fig. 5).

### Effect of vitamin A and *Lactobacillus* on RIG-1 and cytokine expression *in vitro*

**Figure 6:**
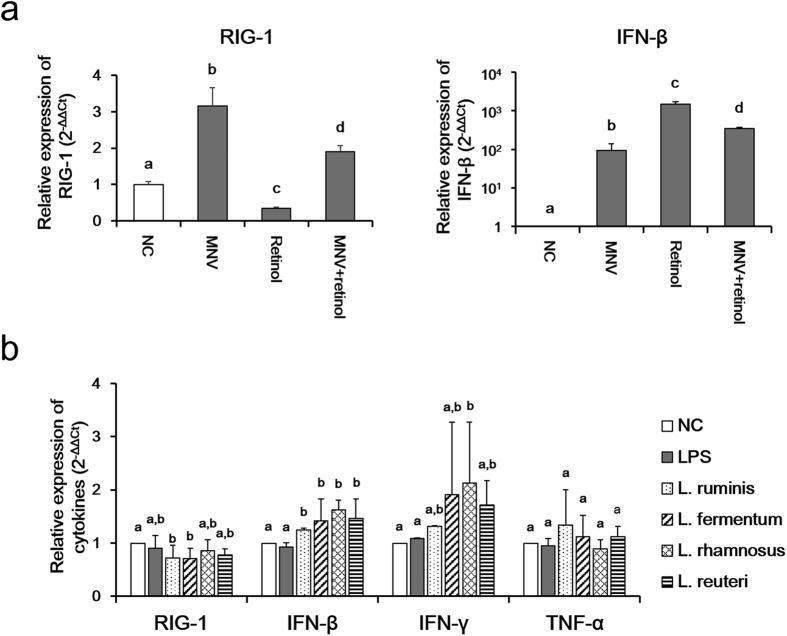
Effects of retinol and *Lactobacillus* strains on the expression of RIG-1 and various cytokines *in vitro.* **(a)** RIG-1 and IFN-β expression in RAW 264.7 cells was measured 24 h after retinol and MNV treatment. **(b)** The effect of *Lactobacillus* strains on RIG-1, IFN-β, IFN-γ, and TNF-α expression in MNV-infected RAW264.7 cells was determined 24 h after *Lactobacillus* treatment. Gene expression was analyzed by SYBR-based quantitative real-time PCR. Different superscript letters indicate significant differences (*p* < 0.05) according to Duncan’s *post hoc* test.

Retinol treatment without MNV infection decreased the expression of RIG-1 in RAW 264.7 cells. In contrast, retinol treatment together with MNV infection significantly increased RIG-1 expression compared to both RIG-1 treatment only and the negative control (Fig. 6a). Moreover, MNV infection significantly increased the expression level of IFN-β, which was further enhanced by retinol treatment (Fig. 6b).

RIG-1 expression in MNV-infected RAW 264.7 cells was significantly decreased by *L. ruminis* and *L. fermentum* (Fig. 6b). IFN-β and IFN-γ expression was also significantly increased by the four *Lactobacillus* strains (Fig. 6b). TNF-α expression was not altered significantly by the *Lactobacillus* strains. LPS showed an antiviral effect with *Lactobacillus* strains did not increase RIG-1, IFN-β, IFN-γ, and TNF-α expression (Fig. 6b).

## Discussion

We demonstrated the inhibitory effect of vitamin A against MNV replication both *in vitro* and *in vivo*. Interestingly, these inhibitory effects occurred directly or indirectly via microbiome changes, particularly on *Lactobacillus* strains in the gut. The prevention of norovirus infection through dietary supplementation has been considered important, as no effective treatment or vaccine against norovirus infection is currently available. This study provides important knowledge regarding the role of particular microorganisms in the gut, such as *Lactobacillus* spp., in preventing acute viral gastroenteritis.

Specific activation of immune responses by RA may induce favorable gut microbiota changes for protection from MNV infection, although a direct effect of RA on gut microbiota composition was not identified. In previous studies, the gut microbiome was determined to be a key modulator for metabolic disorders in TLR knockout mice, including TLR2 and TLR5^11^,^17^, and TLR5-mediated sensing of gut microbiota impacted antibody response to influenza vaccination^18^. Therefore, specific gut microbiota may mediate activation of immune responses by RA to inhibit MNV infection. A previous study reported that MDA5 and TLR3, viral pattern recognition receptors, play distinct roles in the immune response against MNV infection^19^. Moreover, RIG-1 and MDA5 activation is crucial for the immune response to produce IFNs^5^,^20^. Long *et al*. reported that specific chemokines, including monocyte chemoattractant protein-1 (MCP-1), also referred to as chemokine (C-C motif) ligand 2 (CCL2), and interleukin 8 (IL-8), were involved in the immune response against norovirus infection by RA^4^. In addition, retinoid X receptor α (RXRα) plays a key role in innate immunity through the upregulation of chemokines *Ccl6 and Ccl9*^21^. Therefore, these chemokines may mediate antiviral signaling by RA, from viral recognition to inhibition of norovirus.

Interestingly, the gut bacterial community was slightly altered by RA administration, but was significantly altered by MNV inoculation during RA administration. Notably, *Lactobacillus* spp. levels were remarkably increased by MNV inoculation during RA administration. In previous studies, *Lactobacillus* spp. exhibited antiviral effects and alleviated the symptoms caused by rotavirus and influenza viral infections^22^,^23^. Moreover, it has been reported that probiotics, including *Lactobacillus* spp., exert various beneficial effects on human health through their immunomodulatory activities^24^,^25^. In this study, we demonstrated that *Lactobacillus* spp. was enriched by vitamin A intake and significantly inhibited MNV replication.

Over the past several decades, different *Lactobacillus* strains have been isolated from food and human stool and subsequently provided to individuals as probiotics for health purposes^26^,^27^. In this study, we describe *Lactobacillus* strains with antiviral effects, as observed from artificial changes in the gut microbiota. Recent studies demonstrated that gut microbiota was not only influenced by xenobiotics metabolism, but also by medications. Specifically, exposure to certain medications yielded changes in gut microbiota, which resulted in beneficial effects on metabolic disorders^14^,^16^,^28^. Likewise, vitamin A clearly exerted antiviral effects via modulation of gut microbiota, such as *Lactobacillus* spp.

IFN-β plays a key role in the antiviral response mediated by the RIG-1/MDA5 pathway^5^,^20^. It has been reported that certain *Lactobacillus* strains activate IFN-β through induction of RIG-1^29^,^30^. In this study, *Lactobacillus* strains significantly upregulated IFN-β and IFN-γ expression in MNV-infected RAW 264.7 cells. In a recent study, IFN-γ was reported to play an important role in adaptive immunity to MNV infection^31^. Unlike retinol, *Lactobacillus* did not increase the expression of RIG-1, while *L. ruminis* and *L. fermentum* slightly decreased RIG-1 expression in MNV-infected RAW 264.7 cells. Therefore, both IFN-β and IFN-γ are involved in the anti-MNV immune response, in a manner not mediated by the RIG-1/MDA5 pathway. In addition, LPS, which exerts an antiviral effect by inducing the production of various cytokines including IFNs, inhibited the replication of MNV in this study^32^,^33^, but did not increase IFN expression, unlike *Lactobacillus*.

MNV infection was recently shown not to cause major changes in the intestinal microbiota of Swiss Webster and inbred C57BL/6 mice^34^. In contrast, the present study showed that MNV inoculation caused a significant alteration in the gut microbiome; the abundance of Proteobacteria was increased by MNV inoculation, consistent with observations in patients whose gut microbiota was perturbed by human norovirus infection^35^. Moreover, in patients with abundant Proteobacteria, the abundance of Alcaligenaceae was less than in healthy controls^35^. In the *in vivo* study described herein, the decreased abundance of Lactobacillaceae, Alcaligenaceae and Veillonellaceae revealed another significant change in the gut microbiota following MNV infection. Therefore, we believe that the abundance of these bacteria may serve as potential diagnostic and preventive markers for human norovirus infection after specificity issues are addressed.

Most importantly, focus should be placed on norovirus genotypes, which based on previous studies, could be a critical factor in changes in the microbiota, immune responses and viral susceptibility. Norovirus susceptibility in mice is highly associated with MNV genogroups in mouse infection models using six MNV genotypes^36^. Expression profiles of cytokines differ among norovirus genogroups^4^. Moreover, a previous epidemiological study reported that the effect of RA varied depending on the norovirus genotype^3^. Therefore, the gut microbiota composition specific to pathogenic or clinical norovirus infections should be further investigated.

Due to issues with conventional *in vitro* cell culture and the mouse model of human norovirus, MNV has been widely used as a surrogate in pathological and inhibitory studies^37^,^38^. In several recent studies including ours, gut microbiome changes by norovirus were not coincident^34^,^35^. MNV infectivity and pathology differed according to the mouse strain used^19^,^39^. The different results in terms of the effect of MNV on the gut microbiome in our study compared to previous studies was likely due to use of a different mouse model^34^. Disruption of the gut microbiome by human norovirus reported in a previous human study suggested that the gut microbiome is closely related to the immune response to norovirus^35^. The ICR mouse model with MNV-1 used herein may not be the ideal mouse model to study human norovirus. However, further studies will provide insight into the role of the gut microbiome in norovirus infection.

In conclusion, we demonstrated the inhibitory effect of vitamin A on MNV both *in vitro* and *in vivo*, which had only been reported in a human epidemiological study to date. In addition, specific gut microbiota (e.g., *Lactobacillus* spp.) were significantly altered by MNV inoculation during RA administration, and *Lactobacillus* spp. only significantly inhibited MNV replication *in vitro* independent of retinol treatment. We also show that IFN-β is involved in the antiviral immune response. In the future, we expect that the specific modulation of gut microbiota could have an impact on determining functional bacteria for both preventive and medicinal uses.

## Methods

### Cells, viruses and reagents

Huh-7-based NV replicon-bearing cells (HG23 cells) were kindly provided by Dr. Chang^40^. RAW 264.7 cells were maintained in Dulbecco’s Minimal Essential Medium (DMEM) containing 10% fetal bovine serum (Gibco), 10 mM HEPES, 10 mM sodium bicarbonate, 10 mM nonessential amino acids, and 50 μg/mL gentamicin. Retinol (95144; Sigma-Aldrich) was applied to cells following dilution with ethanol. Prior to *in vitro* experiments with retinol, cell viability was confirmed by propidium iodide (PI) staining using a FACScan system (FACSCalibur; BD Biosciences). Prior to all *in vitro* experiments, cell viability against retinol was test using EZ**-**CyTox Cell Viability Assay kit (Daeil Lab Service Co., Ltd, Korea).

### *In vitro* assay to examine MNV inhibition by retinol

Retinol (10, 30, 50, 70, and 100 U/mL) was applied to 80–90% confluent HG23 cells in a 6-well plate. Human norovirus replicon expression was analyzed 24, 48 and 72 h after treatment. Total RNA was extracted using the easy-spin Total RNA Extraction Kit (iNtRON) according to the manufacturer’s instructions. The reaction mixture comprised the AgPath-ID One-Step RT-PCR kit (Ambion). Norovirus GI-specific primers and FAM-labeled GI probes for amplification were described previously^41^. Amplification was performed with a 7300 Real-time PCR System (Applied Biosystems) under the following conditions: reverse transcription (RT) for 10 min at 42 °C, initial denaturation at 95 °C for 10 min, and 45 cycles of amplification, with denaturation at 95 °C for 15 s and annealing plus extension at 60 °C for 60 s. A standard curve was generated using serial dilutions of a norovirus GI-type clone, as described previously^42^. Moreover, inhibition of MNV replication was tested in RAW 264.7 cells. Retinol (10, 30 and 50 U/mL) was applied to 0.01 MOI MNV-inoculated RAW 264.7 cells. MNV replication was analyzed by a plaque assay as described previously^43^ and fluorescence activated cell sorting (FACS) at 24, 48 and 72 h after inoculation.

### Animal model

Twelve-week-old male ICR mice were purchased from KOATECH. (Pyeongtaek, South Korea) and housed in an animal biosafety level 2 (ABL-2) facility at Seoul National University College of Medicine, South Korea. RA (R-2625; Sigma-Aldrich), suspended in corn oil, was administered orally to mice at a dosage of 1 mg/kg/day for eight days. On the seventh day, mice were infected perorally with 5 × 10^6^ plaque forming units (PFUs) of MNV-1 suspended in phosphate-buffered saline (PBS) by oral gavage. The spleen was removed for MNV quantification, homogenized using a PT-2000 E homogenizer (PT-2000 E; POLYTRON) and centrifuged at 10,000 × *g* for 30 min. The resulting supernatants were used for MNV detection.

All experimental protocols in this study were approved by the Seoul National University Institutional Animal Care and Use Committee and were conducted in accordance with the Guide for the Care and Use of Laboratory Animal (SNU-111208-4).

### Analysis of the gut microbiome with and without vitamin A and MNV infection

The gut microbiome was analyzed from mouse whole cecum samples including fecal materials. Cecum samples were homogenized using a homogenizer (PT-2000 E; POLYTRON) and by bead beating with a PowerBead Tube (MO BIO Laboratories Inc.). Total DNA was extracted using the QIAamp^®^ DNA Stool Mini Kit (Qiagen) and QIAcube system (Qiagen). Partial sequences of 16S rRNA genes were amplified according to the 16S rRNA amplification protocol from the Earth Microbiome Project^44^. For each sample, 16S rRNA genes were amplified using the 515F/806R primer set for amplification of the V4 region (515F forward primer: 5′-*AATGATACGGCGACCACCGAGATCTACACTATGGTAATTGT**GTGCCAGCMGCCGCGGTAA***-3′; 806R reverse primer containing a unique 12-base barcode for tagging each PCR product: 5′-*CAAGCAGAAGACGGCATACGAGAT-NNNNNNNNNNNN-AGTCAGTCAGCC **GGACTACHVGGGTWTCTAAT***-3′). Amplified polymerase chain reaction (PCR) products were purified using the UltraClean^®^ PCR Clean-Up Kit (MO BIO Laboratories Inc.). Sequencing of partial bacterial 16S rRNA genes was performed using the MiSeq Reagent Kit V3 (600 cycles) and MiSeq platform (Illumina).

Prior to analysis of the 16S rRNA sequences, BCL files were converted into raw FASTQ files including read1, index and read2 sequences using CASAVA-1.8.2. After preprocessing (quality filtering and trimming steps using FASTX-Toolkit), sequences were assigned to operational taxonomic units (OTUs, 97% identity), and representative sequences were selected using QIIME 1.7.0 software^45^. Next, taxonomic composition, alpha diversity and beta diversity were analyzed. LDA Effect Size (LEfSe) was used to estimate taxonomic abundance and characterize differences between groups^46^. In addition, Phylogenetic Investigation of Communities by Reconstruction of Unobserved States (PICRUSt) was used to predict functional genes in the sampled microbial community based on the KEGG pathway database^47^. A heat map of functional gene abundance was generated using MultiExperiment Viewer (MEV) software (v4.8.1).

### *In vitro* assay to examine MNV inhibition by retinol and *Lactobacillus*

The *Lactobacillus* strain *L. ruminis* SPM 1308 was obtained from Dr. Nam-Joo Ha at Sahmyook University. *L. fermentum* KCTC 3112 was obtained from Korean Collection for Type Cultures (KCTC). *L. rhamnosus* KCTC 18427P and *L. reuteri* KCTC 18428P were additionally isolated in fresh feces of Korean infant. Prior to the inhibition assay, *Lactobacillus* quantity was estimated using optical density (OD). A cell viability test was performed and the concentration of the antibiotic gentamycin (100 μg/mL) was determined, which represents the steady state of *Lactobacillus*. MNV (MOI 0.01) was infected into RAW 264.7 cells for 1 h. Following a media change, *Lactobacillus* (estimated to 1 × 10^5^ CFU by OD), retinol (50 U/mL), and LPS (10 ng/mL) were inoculated for 24 h in MNV-inoculated RAW 264.7 cells. After 24 h, cells were performed a plaque assay to quantify MNV after freezing and thawing twice.

### RNA extraction and gene expression analysis by quantitative real-time PCR *in vitro*

Expression levels of RIG-1, IFN-β, IFN-γ, and TNF-α were analyzed at 24 h after retinol, MNV, and *Lactobacillus* treatment in RAW 264.7 cells. Total RNA was extracted using the easy-spin™ Total RNA Extraction Kit (iNtRON). Then cDNA was synthesized using the High Capacity RNA-to-cDNA Kit (Applied Biosystems) according to the manufacturer’s instructions. To estimate the expression levels of cytokine mRNAs, a QuantiTect^®^ SYBR^®^ Green PCR Kit (204143; Qiagen) and a 7300 Real Time PCR System (Applied Biosystems) were used. The reaction mixture (25 μL) for real-time PCR was composed of 2× QuantiTect SYBR Green PCR Master Mix (12.5 μL), primers (RIG-1: forward CAAAAACCACCCATACAATCAG and reverse CAAATGTGATGTGTACAGGAAG, IFN-β: forward ATAAGCAGCTCCAGCTCCAAG and reverse GTCTCATTCCACCCAGTGCTG, IFN-γ: forward TGAACGCTACACACTGCATC and reverse CGACTCCTTTTCCGCTTCCT, and TNF-α: forward GCCACCACGCTCTTCTGCCT and reverse GGCTGATGGTGTGGGTGAGG, each 50 pmol in 0.2 μL), RNase-free water (11.1 μL), and template DNA (1 μL). GAPDH was used as the internal control.

### Statistical analysis

All features are expressed as the mean ± standard deviation (SD) of each group. Significant differences in the UniFrac distance between groups were analyzed by the Mann–Whitney U-test. In a relative abundance analysis using LEfSe based on the Kruskal–Wallis and Wilcoxon tests, significance was defined as a *p* value <0.05. The threshold on the logarithmic LDA score was set as 2.0–3.0. To quantity the level of mRNA *in vivo* relative to that of an internal control (GAPDH), the 2^−ΔΔCt^ relative quantification method (ΔΔCt = (C_t.Target_ − C_t.GAPDH_)_Group1_ − (C_t.Target_ − C_t.GAPDH_)_Group2_) was used. Statistical significance was assessed by one-way ANOVA followed by Duncan’s *post hoc* test. All statistical analyses were performed using SPSS software ver. 12.0. A *p* value <0.05 was considered to indicate statistical significance.

## Additional Information

**How to cite this article**: Lee, H. and Ko, G. P. Antiviral effect of vitamin A on norovirus infection via modulation of the gut microbiome. *Sci. Rep.* **6**, 25835; doi: 10.1038/srep25835 (2016).
